# Behavioral and Molecular Analysis of Nicotine-Conditioned Place Preference in Zebrafish

**DOI:** 10.1371/journal.pone.0069453

**Published:** 2013-07-24

**Authors:** Ximena Kedikian, Maria Paula Faillace, Ramón Bernabeu

**Affiliations:** 1 Departamento de Fisiología, Facultad de Medicina, Universidad de Buenos Aires, Buenos Aires, Argentina; 2 Instituto de Biología Celular y Neurociencias (IBCN- CONICET), Facultad de Medicina, Universidad de Buenos Aires, Buenos Aires, Argentina; 3 Instituto de Química y Fisicoquímica Biológicas (IQUIFIB)- CONICET, Buenos Aires, Argentina; Tulane University Medical School, United States of America

## Abstract

Studies using mice and rats have demonstrated that nicotine induces a conditioned place preference (CPP), with more effective results obtained by using biased procedures. Zebrafish have also been used as a model system to identify factors influencing nicotine-associated reward by using an unbiased design. Here, we report that zebrafish exhibited putative nicotine biased CPP to an initially aversive compartment (nicotine-paired group). A counterbalanced nicotine-exposed control group did not show a significant preference shift, providing evidence that the preference shift in the nicotine-paired group was not due to a reduction of aversion for this compartment. Zebrafish preference was corroborated by behavioral analysis of several indicators of drug preference, such as time spent in the drug-paired side, number of entries to the drug-paired side, and distance traveled. These results provided strong evidence that zebrafish may actually develop a preference for nicotine, although the drug was administrated in an aversive place for the fish, which was further supported by molecular studies. Reverse transcription-quantitative real-time PCR analysis depicted a significant increase in the expression of α7 and α6 but not α4 and β2 subunits of the nicotinic receptor in nicotine-paired zebrafish brains. In contrast, zebrafish brains from the counterbalanced nicotine group showed no significant changes. Moreover, CREB phosphorylation, an indicator of neural activity, accompanied the acquisition of nicotine-CPP. Our studies offered an incremental value to the drug addiction field, because they further describe behavioral features of CPP to nicotine in zebrafish. The results suggested that zebrafish exposed to nicotine in an unfriendly environment can develop a preference for that initially aversive place, which is likely due to the rewarding effect of nicotine. Therefore, this model can be used to screen exogenous and endogenous molecules involved in nicotine-associated reward in vertebrates.

## Introduction

Tobacco is one of the most commonly used addictive substances; nicotine is its most important neuroactive constituent. Nicotine binds to nicotinic cholinergic receptors (nAChR), ligand-gated ion channels that bind acetylcholine, inducing a cooperative effect with other neurotransmitter systems to modulate synaptic plasticity [1]. Like other addictive drugs, nicotine has profound effects on the midbrain mesolimbic dopaminergic system, increasing excitability and synaptic strength in several brain areas [2], [3]. The dopaminergic signals in this system contribute to associate responses among behaviorally important cues [4]. The highly conserved nature of the rewarding pathway and the universal ability of drugs of abuse to stimulate the nervous system allowed drug-associated reward to be modeled in non-mammalian species [5]–[7].

One of the major challenges in this field is the identification of the cellular and neural circuit-level consequences of reward and relapse in addicts. However, the behavioral screening of drug of abuse effects represents a real bottleneck in this field [8]. A simple vertebrate model for the rapid assessment of cognitive behaviors could be a good solution to find out the effects of nicotine. The zebrafish (*Danio rerio*) has been proposed as an alternative to mammalian models in several fields, including neuroscience [6], [9]–[11]. The zebrafish brain, whereas simpler than the rodent one, is able to control a variety of complex behaviors such as learning, addiction, aggression, as well as social interactions. This species has been used as an animal model for identifying molecules involved in the rewarding effects of drugs [6], [12], [13]. It has been demonstrated that the dopaminergic reward system that projects from the posterior tuberal nucleus to the basal forebrain in zebrafish is reminiscent of the mammalian dopaminergic pathway from the ventral tegmental area to the nucleus accumbens [13], [14]. Moreover, it has been demonstrated that this dopamine projection in zebrafish participates in cocaine reward [6], suggesting that this pathway responds similarly in zebrafish and mammals.

Early studies indicated that nicotine exposure increased the number of nAChR binding sites in the brains of rodents [15]. More recently, it has been confirmed that nicotine up-regulates nAChR without any apparent changes in mRNA levels [16], [17]. Nonetheless, other works demonstrated that nicotine exposure increases mRNA levels of several nAChR subunits in specific brain structures [18], [19]. Several lines of evidence have suggested that phosphorylation of the cyclic AMP response element binding protein (CREB) is highly involved in many forms of experience-dependent plasticity including the rewarding and reinforcing effects of nicotine [20]–[24]. In this context, it was suggested that CREB activity is essential for nicotine CPP [20], [24], [25].

The association between nicotine and environmental cues constitutes a form of conditioning which occurs in humans and other animals. The CPP paradigm is a classical conditioning model that is widely used to investigate the mechanisms underlying context-dependent learning associated with drugs of abuse [20], [26], [27]. Zebrafish have shown Pavlovian conditioning in several tasks including CPP [28]–[30]; zebrafish showed CPP responses to cocaine [6], amphetamine [13], opiates [31], ethanol, and nicotine [12], [32]. An important factor to consider in CPP is the “biased” *vs.* “unbiased” apparatus design. A biased apparatus is one in which animals show a significant preference for one compartment over the other prior to conditioning. In an unbiased box, animals do not show a significant preference for one compartment over the other. Two thirds of the studies in which nicotine induced CPP in rodents used a biased procedure [26], [33], [34]. One report demonstrated nicotine CPP in adult zebrafish by using an unbiased design and showed that CPP persisted following prolonged periods of abstinence [12]. However, previous works had used biased protocols to study different psychostimulants, other than nicotine, which showed positive CPP in zebrafish [6], [13]. Considering that CPP with diverse psychostimulant drugs was efficiently induced by using biased protocols in rodents as well as zebrafish [13], [34], we decided to evaluate nicotine-CPP in adult zebrafish by using a biased protocol. The interpretation of biased CPP results can be difficult. When the drug is paired with the non-preferred side, the possibility of a preference shift due to reduction of aversion could generate misinterpretation of data [27]. Specifically, acute and chronic administration of nicotine can produce either anxiolytic or anxiogenic effects [34].

Nonetheless, we examined whether a biased CPP design, accompanied by a detailed exploration of behavioral measurements [35] and the corresponding control groups, could be useful to study the rewarding properties of nicotine in adult zebrafish. Furthermore, quantification of mRNA level of the most abundant nAChR subunits as well as pCREB protein level was performed in the brain areas related to drugs of abuse effects. The observed expression values correlated with behavioral data.

## Methods

### 1. Animals and Maintenance

Adult zebrafish (*Danio rerio*), approximately 3 months old, were obtained from a local commercial distributor and were maintained according to standard procedures [36]. They were kept in a 100 l tank with a constant 14–10 h light/dark cycle at 28°C and fed twice a day with *Arthemia sp*. and dry food. All fish used in this study were experimentally naïve and were given at least 14 days to acclimatize to the laboratory facility. Afterwards, the animals were moved to the behavioral room and housed in floating chambers (10 cm height × 16 cm top × 13.5 cm bottom × 9.5 cm width) with two animals per chamber. All protocols for animal use, house and care were approved by the Committee on Animal Research of the University of Buenos Aires.

### 2. Drug

Three concentrations of nicotine were dissolved in system water: 15, 30 or 50 mg/l. Concentrations of the drug were calculated by weight of the salt (nicotine hydrogen tartrate salt, Sigma, St. Louis). The pharmacokinetic and pharmacodynamic properties of this drug, including clearance, are unknown for this species. Based on previous studies with zebrafish and mammals [12], [13], [26], [27], we assumed that a period of 20 min of drug exposure should allow the drug to reach and act on the fish brain. Every fish from the nicotine-paired to the white compartment group or the counterbalanced nicotine control group received only one dose of nicotine per day during three conditioning days.

### 3. Conditioning Place Preference (CPP)

#### Apparatus

All experiments were conducted between 9∶00 and 16∶00 h. Our behavioral tank was designed according to Ninkovic and Bally-Cuif [13], with some modifications. The testing tank dimension was 26.5 cm in length, 20 cm in width and 20 cm in depth. Distinct visual cues divided the experimental tank into two halves: one half was colored light-brown and the other half colored white with two black spots placed at the bottom of the tank. Zebrafish clearly preferred the light-brown compartment and avoided the white; therefore it was considered a biased tank. The water level was kept at 12 cm from the bottom of the tank to minimize stress.

#### Pretest

On day 1, after an initial 5·min habituation period in the CPP tank, each fish was tested for baseline place preference by determining the time spent on a given side of the tank over a 10 min period. The preferred compartment was defined as the compartment in which a fish spends most of the time during the pretest [12].

#### Conditioning

One day following the pretest, zebrafish were randomly assigned to one of three treatment groups. Each fish of the nicotine-paired group (putative CPP) was restricted first to the preferred side for 20 min (light-brown side) and then to the white side, where it was exposed to nicotine (15, 30, or 50 mg/l) also for 20 min. Animals in the counterbalanced nicotine control group (nicotine-unpaired group) were first restricted for 20 min to either the white or the brown compartment. Then, they were exposed for 20 min to a single dose of nicotine (15 mg/l) on the first and third day in the brown chamber and on the second day in the white chamber. Zebrafish of the saline-treated group were exposed during the three conditioning days to both sides alternately (20 min in each compartment) without nicotine. The conditioning was run for three consecutive days.

#### Test

On day 5, CPP of each zebrafish was tested in a drug-free environment as in the pretest. We allowed zebrafish to freely swim between compartments and after a 5 min habituation period, the percentage of time spent on each side of the tank was determined for 10 min (test session). During analysis of results, data from the first 2, 5 and 10 min of the test session were compared with the same interval of the pretest session to evaluate changes of preference between both sessions. Changes in place preference were determined by a score [(score % = percentage of the time spent in the non-preferred side during test - percentage of the time spent in the non-preferred side during pretest). Nicotine-induced CPP was assessed on the nicotine-paired group (n = 21) as well as saline (n = 15) and counterbalanced nicotine control animals (n = 12). Control zebrafish were handled in the same way as experimental animals.

### 4. Behavioral Analysis

A camera connected to a computer was placed approximately 1.2 m above the CPP tanks. During pretest, conditioning, and CPP test, zebrafish behavior was recorded and videos were analyzed first by direct observation and then with Noldus Ethovision XT7 software (Noldus Information Technology, The Netherlands, http://www.noldus.com). In a light-brown environment, the contrast between fish and background was higher than with darker colors and conveniently, the video tracking system was able to follow fish without further setup adjustments. The analysis of videos included the following measurements for behavior recording: 1. Time spent in the drug-paired side, the amount of time zebrafish spent in the white compartment. 2. Number and duration of motionless position (stillness for 3 sec or longer). 3. Total distance swum. 4. Average entry duration to the white compartment (time spent in the white compartment divided by the number of entries to the white compartment). 5. Number of transitions to the drug-paired side (number of times the fish entered to the white compartment). 6. Average velocity (distance swum in the brown compartment divided by the time spent in the brown side). Furthermore, during the conditioning session (for three days) zebrafish behavior was recorded to analyze locomotor activity (LA) in both chambers in the presence or absence of nicotine.

### 5. Reverse Transcription (RT) and Polymerase Chain Reaction (PCR)

Zebrafish from CPP experiments were euthanized and three brains were homogenized in buffer from an RNA purification kit (RBC Biosciences, Taiwan) and were considered one sample. Before homogenization, the olfactory bulb, cerebellum, rhombencephalon and most of the optic tectum were removed from each brain. Three independent samples were examined for each treatment and target genes. RNA was quantified and treated with DNAse-I (30 min, 25°C). cDNA was reverse transcribed from RNA with random primers and quantified with a NanoDrop 3300 spectrophotometer (Thermo Fisher Scientific, Waltham, MA). One µg of cDNA was used for standard (45 amplification cycles) or real-time quantitative PCR (qPCR) (RotorGene 6000; Qiagen, Valencia, CA). Specific primers were selected by using Beacon Designer Software (Premier Biosoft International, Palo Alto, CA) from the zebrafish genome reported in the Ensembl database (Table 1). Standard or qPCR products were checked by electrophoresis in 2% agarose gels. No-RT controls, in which the MMLV-reverse transcriptase was omitted, were amplified by qPCR with each primer. No-RT samples showed background fluorescence levels. No-RT and cDNA-containing samples were run in triplicate. β-Actin RNA expression (internal reference gene) showed no significant variations among control and nicotine-treated groups and was amplified in parallel with each gene of interest (target genes).

**Table 1:** Sequence of primers designed to study nicotinic receptor subunit mRNA levels in the zebrafish brain.

Gene	Sense	Antisense
**ß-Actin** (ENSDART00000054987)	TCCCAAAGCCAACAGAGAGAAG	GTCACACCATCACCAGAGTCC
**α4** (ENSDART00000018614)	TGAGAATGTCACCTCCATCAGG	CTTTGCGGTGACTCACTTGACA
**α7** (ENSDART00000051931)	CCGACATCACAGGATACATTGC	GGTAGACGGAATGAGAGGTTCT
**α6** (ENSDART00000031546)	TGTCTGACCCTGTTACTGTGG	CATCAAACTCTGCTGGTGACC
**β2** (ENSDART00000143043)	TGGAGCCCAGAAGAGTTTGATG	CTCCAATGCTGTCGTCTCCTAT

Primers were designed by using Beacon Designer Software (Premier Biosoft International, Palo Alto, CA) from exonic sequences of the zebrafish genome reported in the Ensembl database. The selected primer pairs were specific for the receptor subunit of interest and did not hybridize with other sequences.

### 6. Quantitative Real-time PCR Data Analysis

We searched in the Ensembl and Blast databases nicotinic receptor subunits described in neural tissues. We chose the β2, α4, α7 and α6 subunits because they are the most abundantly expressed in neural tissues in vertebrates. Primers were designed by using Beacon Designer Software (Premier Biosoft International, Palo Alto, CA) from exonic sequences of the zebrafish genome reported in the Ensembl database. The selected primer pairs were specific for the receptor subunit of interest and did not hybridize with other sequences.

Analysis was performed throughout the “Gene expression’s C_T_ Difference” (GED) method [37], which considers individual amplification efficiencies. Briefly, slopes from qPCR kinetic curves obtained as the logarithm of relative fluorescence after n cycles (Rn) plotted against the number of cycles (n) were calculated for each well for β-Actin RNA and target genes. Equation (Eq.) 1 describes the linear range in the exponential phase of the kinetic curve:
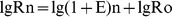
(1)


Ro = initial fluorescence amount and E = PCR efficiency. Within the linear range, we defined a trend-line of 4–5 data points, with the highest possible slope and correlation coefficient, and calculated the efficiency for each reaction (E = 10^slope^-1). Amplification efficiencies for all samples were between 0.93 and 1.07 for reference or interest genes [37]. Samples with efficiencies outside this interval were not included in data analysis.

An arbitrary fluorescence threshold of 0.03 was used to obtain C_T_ values from each curve. We used cDNA from zebrafish that were conditioned with nicotine associated with the white compartment (nicotine-paired) and nicotine administered in both compartments (nicotine-unpaired) as samples of interest. The saline control group was used as the calibrator sample. C_T_ values were previously normalized with β-Actin RNA. We obtained a ratio that indicated the fold-change of mRNA levels of each target gene in nicotine-paired or nicotine-unpaired groups of zebrafish over control values by using the following GED formula:
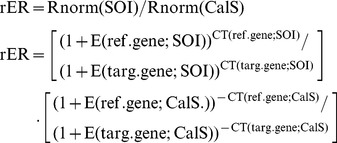
(2)


rER is the relative expression ratio. Rnorm (SOI) and Rnorm (Cal S) are the initial fluorescence amounts in the sample of interest (SOI) and calibrator sample (Cal S), respectively, of the target gene (targ. gene) normalized to the reference gene (ref. gene). The PCR efficiency E was calculated separately and triplicate values were averaged before applying efficiencies in Eq. 2. C_T_ values used in this formula were also averaged from triplicate PCR wells. Each nAchR subunit was run in the same PCR experiment with its own calibrator sample, i.e., a particular calibrator sample was included in Eq.2 for every subunit. For the calibrator sample, relative mRNA expression value equaled 1. Relative values from 3 independent experiments (each consisting of a pool of 3 brains) were reported as mean ± SD.

### 7. Western Blot Assay

Brain tissue was homogenized (the olfactory bulb, cerebellum, rhombencephalon and most of the optic tectum were removed from each brain) in ice-chilled buffer (20 mM Tris·HCl (pH 7.4), 0.32 M sucrose, 1 mM EDTA, 1 mM EGTA, 1 mM PMSF, 10 µg/ml aprotinin, 15 µg/ml leupeptin, 10 µg/ml bacitracin, 10 µg/ml pepstatin, 15 µg/ml trypsin inhibitor, 50 mM NaF, and 1 mM sodium orthovanadate). Aliquots were subjected to SDS/PAGE under reducing conditions. Proteins were electrotransferred onto PDVF membranes for 2 h at 100 V at 4°C. Immunoblots were performed by incubating membranes with an antibody against pCREB (1∶2,000) (Upstate Biotechnology, EMD Millipore, Billerica, MA). Densitometric analysis of the bands was performed with Image Pro plus 6.3 software (Media Cybernetics).

### 8. Statistical Analysis

CPP score for different nicotine concentrations was analyzed using one-way ANOVA. CPP score at different intervals (2, 5 and 10 min) was analyzed by two-way ANOVA (group × time, with time point as repeated measure). During conditioning, the total distance swum in each compartment was analyzed by two-way ANOVA (group × day of conditioning, with day as repeated measure). Behavioral data such as time spent in the white side (sec), average entry duration to white side (sec), number of transitions to the white side, average velocity (cm/sec), total distance swum (cm), and motionless positions (number and duration in sec) were analyzed using two-way ANOVA (group × test, with test as repeated measure). ANOVA statistics were followed by Scheffé *post hoc* comparisons for behavioral analysis. Non-parametric Mann Whitney U test was used for real-time quantitative PCR analysis. pCREB data were analyzed by using one way ANOVA followed by Dunnett’s test. Data were presented as the mean±SEM and significance was set at p<0.05. All data analysis was computed by using Stat View 5.0.1 software.

## Results

### 1. Exposure to Nicotine Induced Biased CPP in Zebrafish

**Figure 1:**
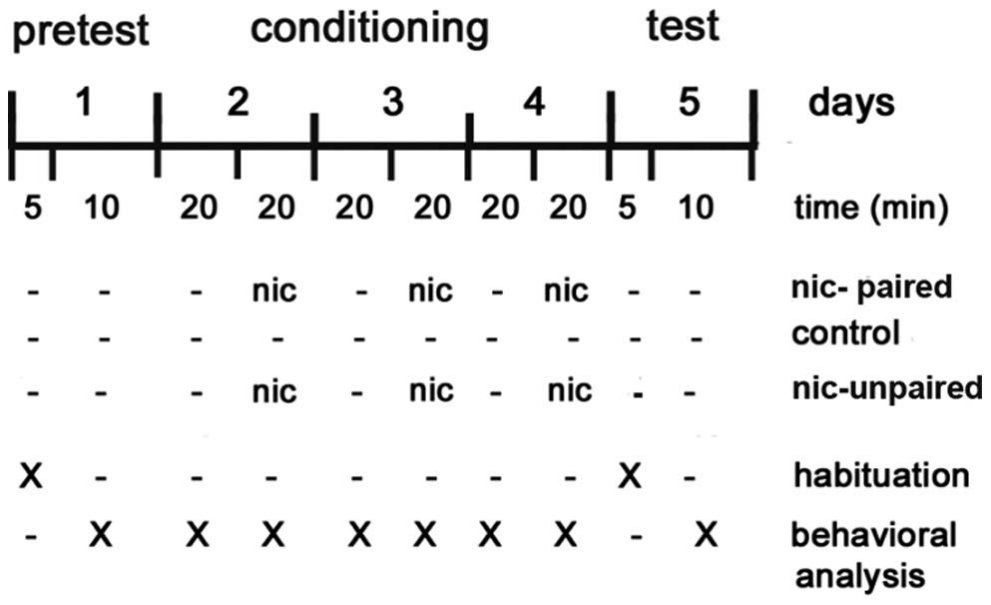
Schematic diagram showing the experimental CPP procedure. On day one (pretest), each zebrafish was tested for baseline preference for each side of the tank (light brown or white). On days two, three and four, zebrafish were individually restricted first to the brown side for 20 min and consecutively to their non-preferred side (white side), where they were exposed to nicotine (nicotine-paired group) or saline (control group) for 20 min. For the counterbalanced nicotine-unpaired control, animals were restricted to the brown or white compartment for 20 min and then exposed to nicotine alternatively in the brown (days 1 and 3) and the white (day 2) compartment also for 20 min. On day five, the place preference of each zebrafish was measured by determining the percentage of time spent on each side of the tank over a 5 min test period, following a 5 min interval for habituation. Behavioral analysis was performed during the five days as indicated by crosses.

**Figure 2:**
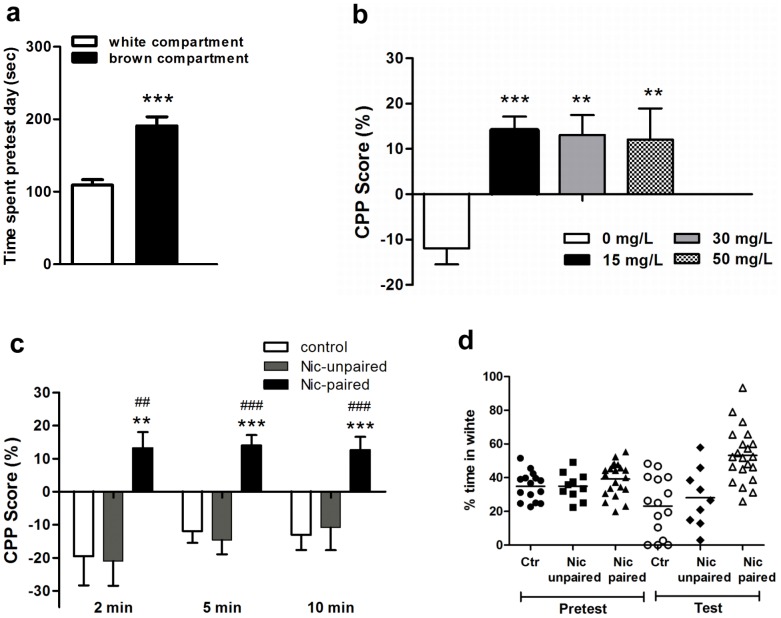
Baseline preference and conditioned place preference (CPP). Figure ***a)*** Amount of time spent by naïve zebrafish in the white or brown compartment of the CPP tank during 5 min in a drug-free pretest. ***b)*** CPP establishment at different nicotine concentrations 0 (control), 15, 30 and 50 mg/L. CPP score was calculated as % of time spent in the drug-paired side after drug exposure (test) minus % of time spent on the drug-paired side before drug exposure (pretest) over a 300 s time period. ***c)*** Shows 15 mg/L nicotine-CPP scores for nicotine-paired, nicotine-unpaired and saline control groups at different time points after a 5 min interval of habituation. ***d)*** Shows the percentage of time spent in the white compartment during pretest and test sessions for individual zebrafish from the 3 groups. Data are presented as mean ± SEM. 0 mg/L (control) n = 15; nicotine-unpaired n = 12; nicotine-paired: 15 mg/L n = 21, 30 mg/L n = 12, and 50 mg/L n = 12. ** *p*<0.01, ***p<0.001 nicotine-paired *vs*. control and ## *p*<0.01, ### *p*<0.001 nicotine-paired *vs* nicotine-unpaired.

Figure 1 shows the final schedule selected for nicotine-CPP. Figure 2a depicts the average time spent in each compartment during 5 min (in white 114.48±5.28 *vs.* in brown 185.51±5.87 sec). Analysis of data revealed that animals showed a significant preference for the brown compartment (*t*(64) = 51.5, *p*<0.001). Interestingly, a similar relationship was found at 2 (49.78±5.11 *vs.* 70.21±4.76 sec) and 10 min (222.03±8.63 *vs.* 377.97±9.31 sec). Based on previous studies performed by Kily et al. by using an unbiased protocol [12], we determined the concentration and time of exposure to the drug at which zebrafish exhibited CPP in response to nicotine. However, we used the environment which was previously employed with amphetamine and cocaine (a biased design) [6], [13]. One-way ANOVA revealed significant differences (*F*_3.57_ = 8.343, *p*<0.0001) between control and CPP animals (figure 2b), showing a more significant effect at 15 mg/l of nicotine in CPP (*p*<0.001) compared with 30 and 50 mg/l (*p*<0.01). Next, we evaluated CPP scores at different time points using 15 mg/l of nicotine. Figure 2c shows the cumulative time that zebrafish spent in the nicotine-paired side during 2, 5 and 10 min, following a 5 min interval of habituation to the tank. There were significant differences when we analyzed the 3 series of data (*p*<0.01 for 2 min and *p*<0.0001 for 5 and 10 min). Figure 2d depicts the percentage of time spent in the white compartment during pretest and test sessions for individual zebrafish from the 3 groups (control, nicotine-unpaired and nicotine-paired groups). Taking together these results, we selected 15 mg/l of nicotine, which was the lowest nicotine concentration that showed the highest CPP score. Because high concentrations of nicotine induced toxicity in zebrafish and its metabolism is unknown, we considered that repeated exposure may lead to either toxic build up of the drug in the fish or the development of tolerance, both effects with possible consequences on CPP response. Moreover, a 5 min interval for drug exposure was chosen because it was a period of time that was sufficient to avoid unspecific effects on locomotor activity associated with zebrafish manipulation, but was not sufficient for observing a reduction in the LA due to familiarization with the tank.

### 2. Behavioral Characterization of Nicotine Biased Place Preference in Zebrafish

**Figure 3:**
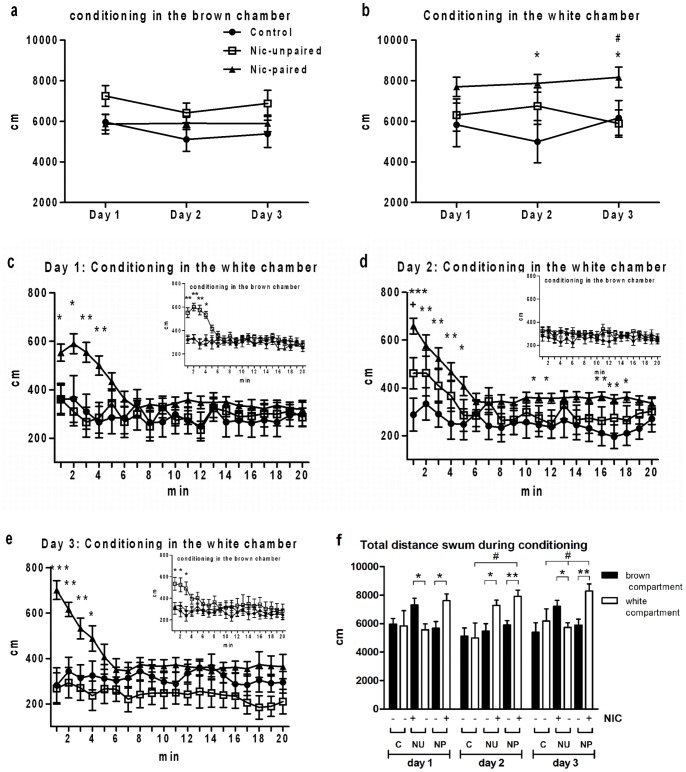
Total distance swum in the brown or the white compartment during each conditioning day. *****a)***** Shows the total distance swum in the brown compartment on days 1, 2 and 3 of the conditioning session by each of the three groups of zebrafish (saline, nicotine-unpaired, nicotine-paired). ***b***) Displays the total distance swum in the white compartment on days 1, 2 and 3 during conditioning. ***c) to e)*** The total distance swum was measured minute-to-minute during the whole conditioning session (20 min) and plotted for days 1, 2 and 3 (c, d and e), in the white chamber and in the brown chamber (upper right inset in c, d and e). ***f)*** Comparison between saline control and nicotine-treated groups in distance swum each day of conditioning in the brown and white compartments. During conditioning, nicotine-paired zebrafish were exposed to nicotine in the white compartment. The nicotine-unpaired group was exposed to nicotine in both sides (days 1 and 3 in the brown and day 2 in the white). Data are presented as mean ± SEM. *p<0.05; **p<0.01 and ***p<0.001 between control and nicotine-paired groups. ^#^ *p*<0.05 between nicotine-unpaired and nicotine-paired animals in the white compartment. +*p*<0.05 between nicotine-unpaired and control. Control (C) = saline n = 15; Nic-unpaired (NU) = counterbalanced nicotine n = 12; and Nic-paired (NP) = nicotine-associated to the white compartment n = 21.

Once concentration and time of exposure to the drug were fixed, we characterized some specific responses for identifying preference-related behaviors to nicotine-conditioning in zebrafish. LA was measured for each conditioning day in both compartments in the three groups (Figures 3a–f). Two-way ANOVA revealed significant differences among groups per day of conditioning (*F_2,24_* = 6.252; *p*<0.05). Scheffé *post hoc* comparisons showed significant differences in the distance swum between saline control and nicotine-paired groups during the second day (*p*<0.05) and between both control groups (saline and nicotine-unpaired) and the nicotine-paired group on the third day of conditioning (*p*<0.05), which was observed exclusively in the white compartment (figures 3b and f). Moreover, the distance swum for nicotine-paired zebrafish was significantly longer inside the white than in the light-brown compartment (*p*<0.05 for day one and *p*<0.01 for days two and three) during the three days of conditioning (figure 3f). In contrast, no significant differences between saline and nicotine-unpaired control and the nicotine-paired group were found during conditioning in the light-brown compartment (figures 3a and f). However, statistical differences in the distance swum were found in nicotine-unpaired compared with saline-treated animals when zebrafish were exposed to nicotine (days 1 and 3 in the brown and day 2 in the white compartment, *p*<0.05). Similar values for the distance swum in the light-brown compartment were found in animals without nicotine treatment in the white compartment (figure 3f). To better evaluate the effect of nicotine, we quantified the distance swum during conditioning by using a minute-to-minute interval (figures 3c–e). Zebrafish exposed to nicotine showed increased LA for the first 5 min; after that, both nicotine-treated (paired and unpaired) and saline groups displayed the same values of distance swum (except for small but significant differences observed on day 2 in the nicotine-paired group during the last 10 min of the interval). Analysis of the LA by using a minute-to-minute interval inside the light-brown chamber, during the three days of conditioning, showed no significant differences between the saline control and nicotine-paired groups (figures 3c–e, upper right inset). However, the nicotine-unpaired group showed a significantly higher LA, compared with both nicotine-paired and saline groups for the first 5 min during days 1 and 3 of conditioning when it was exposed to nicotine in the brown chamber, whereas nicotine-paired and saline groups were not exposed to the drug.

**Figure 4:**
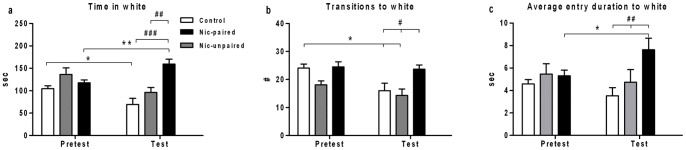
Baseline (pretest) and test values of behavioral parameters in the non-preferred compartment in nicotine-CPP. CPP was performed by using 15 mg/L of nicotine during conditioning and the time course examined was of 5 min following 5 min habituation during the pretest and test sessions. Panel ***a)*** shows the time spent in the white compartment, and ***b)*** the number of transitions to the white compartment. ***c)*** This graph shows the average entry duration to the white compartment. Data are presented as mean ± SEM. Control: saline n = 15, Nic-unpaired n = 12, and Nic-paired n = 21. *p<0.05 and **p<0.01 between pretest and test and ^#^ *p*<0.05, ^##^ *p*<0.01 and ^###^ *p*<0.001 between controls (saline and Nic-unpaired) and Nic-paired. Control: saline; Nic-unpaired: counterbalanced nicotine treatment and Nic-paired: nicotine treatment associated to the white compartment.

Next, the effect of nicotine on CPP was evaluated by analyzing behavioral changes before (pretest) and after (test) conditioning. Figure 4a shows a significant increment in the total time spent in the white compartment, as a CPP-positive parameter, when both control groups and the nicotine-paired group were compared during the test session (two-way ANOVA *F_2_*_,44_ = 27.121; *p*<0.0001). Moreover, a significant decrease in the total time spent inside the white compartment in the control group (*p*<0.05) and an increase in this parameter in the nicotine-paired group (*p*<0.01) were observed between pretest and test sessions. Furthermore, control groups (saline and nicotine-unpaired) displayed a reduced number of transitions to the white compartment in the test compared with the pretest (*p*<0.001 and *p*<0.01, respectively). A similar significant decrement was observed for both control groups compared with nicotine-paired animals during the test session (figure 4b; *p*<0.05). In figure 4c, the average entry duration to the white side was analyzed. This parameter exhibited significant differences among treatments (*F_2_*_,44_ = 7.427; *p*<0.01). Nicotine-paired compared with control groups showed a significant increase in the average entry duration during the test session (*p*<0.01). The nicotine-paired group also showed a significant enhancement of this parameter during the test compared with the pretest session (*p*<0.05).

**Figure 5:**
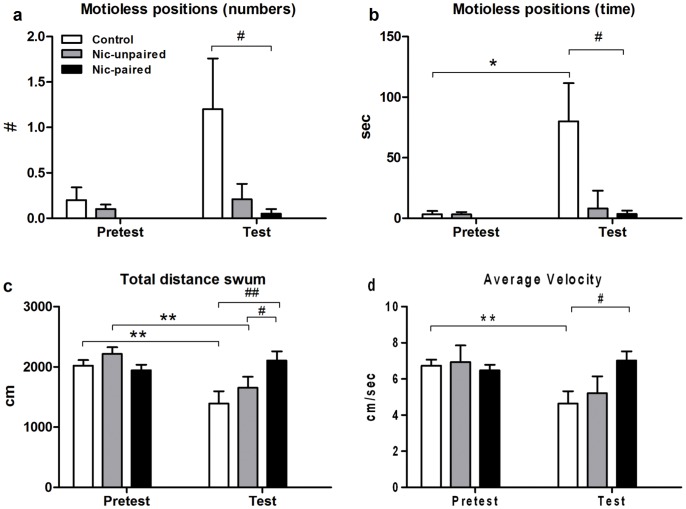
Behavioral effects of nicotine (15 mg/L) on zebrafish in the CPP tank. Analysis of behavioral parameters was performed before and after nicotine conditioning on a 5 min test. Figures ***a)*** and ***b)*** show the number and duration of motionless positions in the brown side. Figure ***c)*** the total distance swum and ***d)*** the average velocity, which were calculated by using Ethovision XT7. Data are presented as mean ± SEM. Saline control n = 15, counterbalanced control n = 12, and nicotine-paired n = 21. *p<0.05 and **p<0.01 between pretest and test and ^#^ *p*<0.05 and ^##^ *p*<0.01 between controls and nicotine-paired group. Control: saline; Nic-unpaired: counterbalanced nicotine treatment and Nic-paired: nicotine treatment associated to the white compartment.

Figures 5a and b show the number and duration of motionless positions. Significant differences in the number (*F_2_*_,44_ = 6.752; *p*<0.05) and duration (*F_2_*_,44_ = 8.852; *p*<0.01) of motionless positions were observed among groups. The number of immobile states was significantly increased (p<0.05) during the test session in control animals (figure 5a). Moreover, we found a significant increase in motionless position duration in control zebrafish in the test compared with the pretest session (*p*<0.05). The saline control showed significant differences (*p*<0.05) compared to nicotine-paired but not to nicotine-unpaired group during the test session. It is important to mention that no motionless positions were observed in the white compartment.

Considering these and previous results, we evaluated stimulant effects of nicotine by measuring the total distance swum for each fish during pretest and test sessions. Figure 5c shows significant differences in the total distance swum among groups (*F_2_*_,44_ = 10.743; *p*<0.01). A significant decrease in this parameter was observed in both control groups between pretest and test sessions. Furthermore, control and nicotine-unpaired groups showed significantly lower values than nicotine-paired animals (*p*<0.01 and *p*<0.05, respectively) in the test session. Finally, in figure 5d, the average velocity was analyzed in each compartment during pretest and test sessions. Two-way ANOVA revealed significant differences among groups in the test session (*F_2_*_,34_ = 11.003; *p*<0.01). A significant reduction in the average velocity was found in the control and nicotine-unpaired groups (p<0.01 and p<0.05) in the test session compared to the pretest control group. The saline control group was significantly different from the nicotine-paired group during the test session (*p*<0.01).

### 3. mRNA Expression Levels of nACh Receptor Subunits in the Brain of the Adult Zebrafish

**Figure 6:**
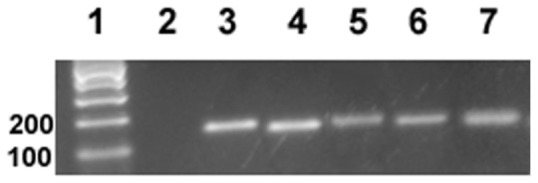
mRNA expression of nAChR subunits in the zebrafish brain portions that contain reward pathway related structures. The primers designed (see table 1) were used in a standard RT-PCR to determine if zebrafish brain expressed specific subunits of nAChR. PCR products were separated in a 2% agarose gel. Lanes 1: DNA marker; 2: no RT; 3: β-actin; 4: α6; 5: β2; 6: α4 and 7: α7.

**Figure 7:**
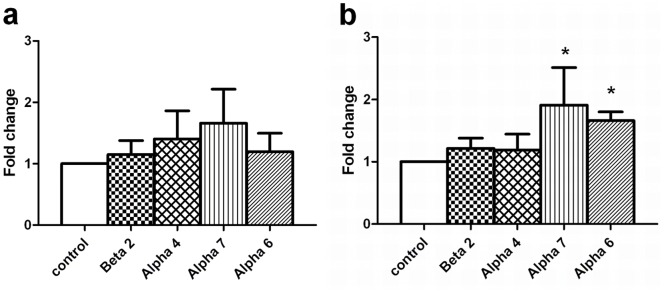
Nicotinic receptor subunit mRNA expression level was identified by RT-qPCR in the zebrafish brain. Panel a) represents fold change of mRNA expression for each nicotinic receptor subunit tested in saline control (white bar) and nicotine-unpaired (counterbalanced) group of zebrafish (patterned bars). Panel b) depicts mRNA expression of nicotinic subunits examined in saline control (white bar) and nicotine-paired (associated to the white chamber) groups of zebrafish (patterned bars). RNA was purified from pools of three brains each obtained from control or nicotine-treated animals. Fold change represents mean ± SD, from three independent experiments of the relative expression ratio, that is the initial fluorescence amount in the sample of interest (nicotine-paired or nicotine-unpaired zebrafish brain) relative to a calibrator sample (saline control zebrafish brain) normalized to an internal reference gene (β-actin). Control value for each type of subunit equals 1 after normalization. Fold change was calculated by “Gene expression’s C_T_ Difference” method (see Methods). *p<0.05 by using non parametric Mann Whitney test.

In order to evaluate whether the nicotine treatment in paired or unpaired conditions in zebrafish is associated to changes in the level of expression of the nicotinic receptor subunits, we examined mRNA expression levels of the most frequently expressed nAChR subunits in the brain following the test session. By using standard RT-PCR, we found that β2, α4, α7 and α6 subunit mRNAs were expressed in adult zebrafish brain structures representative of the mesolimbic pathway (see Methods section) (Figure 6). Then, we assessed the expression level of these four nAChR subunits by quantitative real-time PCR. The mRNA expression level of α7 and α6 receptor subunits in nicotine-paired zebrafish brains were 1.91 and 1.66 times higher than these receptor subunit mRNA levels in the saline control group (*p*<0.05). In contrast, no significant differences were observed between subunit mRNA levels in nicotine-unpaired zebrafish brains and saline control values. β2 and α4 receptor subunit mRNA levels from zebrafish brains were not statistically different from saline control animals under any condition (Figure 7).

### 4. pCREB Levels in a Portion of the Adult Zebrafish Brain Containing Rewarding-related Structures

**Figure 8:**
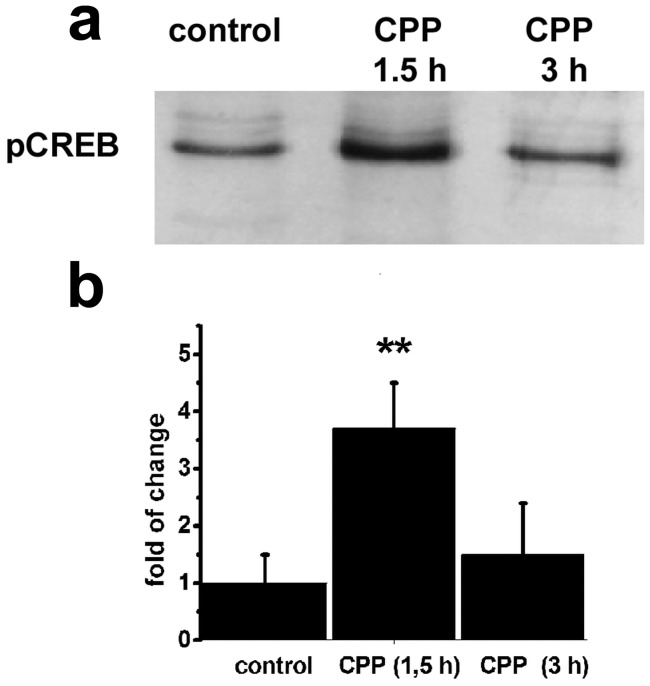
Levels of pCREB protein in the zebrafish brain following nicotine-CPP. a) Western blot of pCREB in control and nicotine-paired zebrafish (CPP positive) that were euthanized 1.5 or 3 h following CPP test. b) Quantitative densitometry analysis of the positive band obtained in (a) expressed as a ratio relative to control zebrafish euthanized 1.5 h following CPP test. N = 6 per group, **p<0.01 Dunnett’s test after ANOVA.

We evaluated the levels of pCREB in control and CPP-positive zebrafish brain portions containing structures of the reward pathways. pCREB was selected considering that it has been described as a good marker for evaluating changes in neuronal activity in nicotine-treated animals [17], [36]. Because the phosphorylation of CREB is a transient mechanism, levels of pCREB were assessed 1.5 and 3 h following the CPP protocol and test. Figure 8 (a) shows an image of the blot obtained by using a specific antibody against CREB phosphorylated at serine 133 and (b) the quantification of band densities showed in (a). A significant increase in the level of pCREB was observed at 1.5 h after nicotine-CPP, but not at 3 h post-test (p<0.01) compared with saline control zebrafish.

## Discussion

In the present study, we have examined a biased protocol for studying nicotine-CPP in zebrafish together with a detailed behavioral analysis. Measurements of mRNA expression of several subunits of nicotinic receptors and the brain levels of pCREB were also performed to evaluate the participation of these molecules, which have been described as markers of nicotine-CPP in mammals [19], [20], [38]. Animals receiving nicotine in the white compartment spent more time in that compartment on the test day compared to saline control and nicotine-unpaired animals, which provided evidence of the establishment of nicotine CPP in zebrafish.

Zebrafish were previously used to test different drugs of abuse, such as cocaine, amphetamine, morphine, ethanol and nicotine, in CPP protocols [6], [12], [13], [30], [32], [39]–[41]. In particular, nicotine-CPP was formerly examined by using an unbiased protocol [12]. However, in this study we evaluated the rewarding properties of nicotine in zebrafish by using a biased protocol according to the following reasons. In previous studies, a biased tank was used to test the rewarding effects of stimulants such as amphetamine and cocaine [6], [13]. In fact, in biased protocols, animals spend a substantial amount of time in the initially non-preferred chamber (even longer than in the naturally preferred side), which likely indicates the strength of the rewarding properties of this particular drug by forcing the animal’s permanence in an aversive environment. Furthermore, some authors suggested that nicotine-CPP is more effectively induced by using a biased protocol [33], [34], [42].

Our design showed that zebrafish clearly preferred the light-brown compartment; therefore, the initial aversion for the white environment was overcome by the rewarding effect of nicotine. This preference did not occur with the nicotine-unpaired group, in which nicotine effect was not associated with a particular environment. Additionally, the light-brown background was better for the tracking system to accurately follow animal’s movements, allowing the performance of several behaviors to be analyzed. According to our experience, both methodologies (biased and unbiased) can be successfully used in rodents [20], [43] as well as zebrafish (present and Kily et al. results [12]).

Exposure for three days to nicotine concentrations of 15, 30 and 50 mg/L induced a significant increase in time spent in the drug-paired side compared with control fish, indicating a consistent reinforcement response. 50 mg/L was more than 3 times higher than the lower dose examined; this observation opens the possibility to evaluate a widest range of nicotine concentrations that can induce CPP in zebrafish. These studies are not suitable for rats, because a dose two times higher than the one that induces CPP provokes strong aversion [20], [43]. Another important point to consider is that experimental rats usually represent a single strain (Wistar, Sprague-Dawley, and Long Evans), whereas for zebrafish, we used a pool of genetically variable wild type animals, which could make these results pertinent to a more diverse population. We also obtained reproducible reinforcing effects measuring CPP scores at 2, 5 and 10 min after 5 min habituation to the tank. This result suggests that zebrafish could be tested for positive CPP for different intervals of time, offering the possibility of pharmacological intervention by adding compounds into the water of the CPP tank.

The finding that zebrafish in the nicotine-paired group spent more time in the non-preferred compartment in the test session, compared to saline control and nicotine-unpaired animals, provided evidence that nicotine did not cause a reduction in aversion to the white compartment, suggesting that nicotine produces preference through its rewarding effects, as it does in rodents [34].

The behavior of each zebrafish was analyzed during conditioning. It was observed that, in nicotine groups (paired and unpaired), the locomotor activity was increased when the animals were exposed to nicotine, demonstrating that this drug had an excitatory effect on zebrafish activity.

We detected significant intra-group differences between the brown and white compartments when animals were exposed to nicotine and inter-group differences on the second and third day of conditioning in the white compartment, between nicotine-paired and control fish, suggesting that nicotine may produce a different effect on LA when delivered in the non-preferred environment. Interestingly, we observed that LA in nicotine exposed animals during conditioning was significantly increased within the first 5 min of exposure to the drug. These changes might not be associated with stress related to the aversive environment, since control zebrafish showed no LA changes in either the initial 5 min or the whole conditioning session in the white side. Previously, the involvement of nAChR desensitization in nicotine-induced decrease of LA has been observed [44], [45]. Therefore, following 5 min of nicotine exposure, at least some nicotinic receptors might desensitize with the consequent reduction in LA.

As previously pointed out, in order to determine addiction-like behaviors, it is relevant to characterize various components of the behavioral response [8], [35], [46]. On the other hand, partial or wrong conclusions could be obtained by measuring molecular parameters, such as gene expression or posttranslational protein modifications, if behavior is not deeply described and analyzed. Therefore, we evaluated relevant behavioral parameters such as permanence in a particular compartment, transitions and average entry duration to the drug-paired side, motionless states (number and duration), average velocity and total distance swum, all of which provided evidence of the behavioral state of the zebrafish as a consequence of drug exposure [35], [47]. In parameters such as permanence in and average entry duration to the white chamber, it was confirmed that the nicotine-paired group displayed a differential behavior in the non-preferred side compared to controls, and also between pretest and test, which correlates with the observation that only nicotine associated to the non-preferred compartment induced the preference. Control zebrafish showed lower scores in the test session compared to the pretest session in two of the parameters measured. The decrease in permanence in and transitions to the aversive side suggested that control zebrafish chose to stay in the less aversive side, confirming their initial preference. The observed decrease in total distance swum for both saline control and nicotine-unpaired groups between pretest and test sessions suggested that nicotine *per se* had no effect on this parameter. In contrast, nicotine-unpaired animals did not show a significant decrement in this parameter, suggesting that the association nicotine-environmental cue did affect the LA. In “motionless states”, the meaning of the higher score found only in the saline control group during the test session is uncertain. Some studies have reported increases in zebrafish naïve exploratory behavior and decreases in their freezing behavior during a 6-min interval in a novel tank test [35]. Immobile behavior could indicate fear; however, in this study, zebrafish exclusively from the saline control group showed this type of behavior during the test session. These animals had been exposed several times to the conditioning tank without nicotine and the motionless postures were observed in the less aversive side. In contrast, nicotine-unpaired animals showed no significant differences with nicotine-paired zebrafish, which suggests that nicotine *per se* affected this parameter. Considering that no evidence for fear or anxiety was found, we believe that the observed long periods of motionless posture in the light-brown chamber were principally associated with habituation to the initially preferred side of the tank [47], [48].

The rewarding effect developed during conditioning is determined by the association between the drug and the compartment where it was delivered, which gave obvious results, as there was a significant increase in the time spent in the drug-associated compartment and entry duration to the white side between pretest and test sessions in the nicotine-paired group. In contrast, these behaviors were absent in control groups with or without nicotine exposure. It is important to remark that, particularly with “transitions to white compartment”, “total distance swum”, and “average velocity”, no significant differences between pretest and test sessions were observed in the nicotine-paired group. We consider that this is not due to an effect of nicotine itself, because nicotine-unpaired control animals showed a significant decrement in total distance swum in the brown compartment between pretest and test sessions. LA was increased in the presence of nicotine and during a brief period of time (5 min). Considering that pretest and test are nicotine-free sessions, it is possible that long-lasting nicotine effects after conditioning kept LA elevated only in the nicotine-paired group, which is similar to that which was observed when zebrafish were exposed to a novel environment during the pretest session. In contrast, the nicotine-unpaired group, even if exposed to the same amount of drug, did not keep LA at the same level as in the pretest session when novelty was an issue.

Considering that nicotine-paired zebrafish performed positive CPP, we evaluated whether the four most abundant nicotinic receptor subunits were differentially expressed in zebrafish brain in control and nicotine-paired groups. Repeated nicotine pretreatment induces up-regulation of nicotinic receptors [17], [49]. Changes in β2, α4, α6 and α7 subunits have been suggested to contribute to this receptor up-regulation [50], [51]. The present study has revealed that the expression of α7 and α6 genes in reward brain areas was modestly but significantly increased in zebrafish that performed nicotine-CPP. In contrast, β2 and α4 subunits were not affected by nicotine-CPP at a transcriptional level. Noteworthy, no significant changes were observed in zebrafish brains exposed to nicotine for three days in an unpaired manner, which suggests that the observed increase in α6 and α7 subunits might be involved in the place preference shift induced by nicotine. This is the first report identifying quantitative changes in specific nAChR subunits after nicotine CPP in zebrafish. Nevertheless, the observed changes after test and around 24 h following the last nicotine exposure might not be causally related to CPP; therefore, further studies will be necessary to elucidate this issue. Different groups have suggested different subunits of nicotinic receptors as the most important components in nicotine dependence [2], [3], [19], [38], [52]. In mice, the β2 subunit but not α7 is critically involved in nicotine reward [25]. Moreover, targeted genetic deletion of the α4 subunit from dopaminergic neurons increased sensitivity to nicotine-induced locomotor depression in mice [2]. On the other hand, it was found that the α7 subunit potentiates nicotine reinforcing action in the VTA by regulating DA outflow in the NAcc [53]. Furthermore, α6 was suggested as the principal subunit expressed in rat striatum involved in nicotine addictive behaviors [54]. The sequence homology data provides evidence of conservation of nAChR between zebrafish and mammals [32]. However, antagonists of the α4β2 and α7 nAChR that are effective in mice did not show similar effects in zebrafish, suggesting that nAChR subunit composition involved in nicotine reinforcing effects could be different in zebrafish [55].

To better characterize the effect of nicotine on CPP at a molecular level, we measured the phosphorylation state of CREB in brain portions containing reward pathways from conditioned animals. pCREB was increased in nicotine-CPP zebrafish compared to control animals when they were sacrificed at 1.5 h but not at 3 h post-test. These results demonstrated that pCREB, in the brain of zebrafish that showed a conditioned place preference to nicotine, exhibited a short-term transient activation which might be sufficient to induce CPP. Our results agreed with previous studies in mammals [20], [24], [25], which suggested that pCREB is a good marker for the evaluation of drug preference in rats and mice. Our results extended this observation to zebrafish.

Zebrafish offer the advantage, compared to mammals, that pharmacological studies can be performed without any invasive stressful intervention, such as i.p. injections. Moreover, exposure to the drug can be continuous, since the animal can receive the drug for several minutes to hours, helping to determine the pharmacokinetic values of the drug [10], [56].

In summary, our results indicated that zebrafish is a good model for screening the rewarding properties of nicotine. We demonstrated that these animals showed a clear preference for the aversive environment associated with the drug, which was indicated and supported by several behavioral parameters. Changes in nicotinic receptor subunit expression as well as transcription factor activation were also described, which may contribute to the behavioral changes. It is possible that animals that showed increased baseline anxiety (expressed throughout increased initial preference for the brown side) are more sensitive to the rewarding properties of nicotine. We are planning to examine whether this first response to the biased tank is a predictor of vulnerability to nicotine, as we have previously demonstrated in rats [57]. We hope that this information eases the interpretation of results from nicotine CPP studies and sheds light on additional questions pertaining to the mechanisms involved in nicotine preference using the robust assay that we have established in the present study.
